# The Ethnological Phenomenon

**Published:** 1880-09

**Authors:** J. C. Beckham

**Affiliations:** Zebulon, Pike Co., Ga.


ARTICLE VII.

### THE ETHNOLOGICAL PHENOMENON.

ZEBULON, PIKE CO., GA., Aug. 14, 1880.

*Prof. Harvey L. Byrd, M. D.*

DEAR SIR:—Please excuse me for addressing this note to you, I accidentally got hold of *The Independent Practitioner*, published in Baltimore, in which is your communication on an "Ethnological Phenomenon," page 375, in which you state "that whether the offspring of caucasian women and negro men, present the same peculiarity, I have had no means of knowing." Thirty-five years experience has given me the opportunity of settling that point. In one case, when white women were present and the subject of the child's parentage was discussed, I called their attention to *its scrotum as an evidence* that it was a mulatto. Another thing I have noticed, and which will *invariably* hold good, children of white women, and negro men, are much darker, and more like the negro race, than children of negro women and white men! I believe, and indeed, I have had opportunity of noticing *this mark* to be manifest *in those who were not known to be contaminated with negro blood!* I am satisfied it is like the brand of Cain, *Indelible*, and it is not only in the *octoroon* but in *perpetual* generations that *this mark* will follow.

Excuse me for addressing this letter to you? But in my long experience, and noticing *this particular thing*, I am satisfied that God has purposely placed it there to keep the Caucasian race free from adulteration.

Very respectfully yours,

J. C. BECKHAM, M. D.

